# Elastin Peptides Signaling Relies on Neuraminidase-1-Dependent Lactosylceramide Generation

**DOI:** 10.1371/journal.pone.0014010

**Published:** 2010-11-16

**Authors:** Anthony Rusciani, Laurent Duca, Hervé Sartelet, Aurore Chatron-Colliet, Hélène Bobichon, Dominique Ploton, Richard Le Naour, Sébastien Blaise, Laurent Martiny, Laurent Debelle

**Affiliations:** 1 Laboratoire Signalisation et Récepteurs Matriciels (SiRMa), UMR CNRS 6237, Université de Reims Champagne Ardenne, Faculté des Sciences, Reims, France; 2 Laboratoire Médicament, Dynamique Intracellulaire, Architecture Nucléaire (MéDIAN), UMR CNRS 6237, Université de Reims Champagne Ardenne, Faculté de Pharmacie, Reims, France; 3 Laboratoire d'Immunologie et de Microbiologie, EA 4303 Inflammation et Immunité de l'appareil respiratoire, Faculté de Pharmacie, Reims, France; Johns Hopkins School of Medicine, United States of America

## Abstract

The sialidase activity of neuraminidase-1 (Neu-1) is responsible for ERK 1/2 pathway activation following binding of elastin peptide on the elastin receptor complex. In this work, we demonstrate that the receptor and lipid rafts colocalize at the plasma membrane. We also show that the disruption of these microdomains as well as their depletion in glycolipids blocks the receptor signaling. Following elastin peptide treatment, the cellular GM_3_ level decreases while lactosylceramide (LacCer) content increases consistently with a GM_3_/LacCer conversion. The use of lactose or Neu-1 siRNA blocks this process suggesting that the elastin receptor complex is responsible for this lipid conversion. Flow cytometry analysis confirms this elastin peptide-driven LacCer generation. Further, the use of a monoclonal anti-GM_3_ blocking antibody shows that GM_3_ is required for signaling. In conclusion, our data strongly suggest that Neu-1-dependent GM_3_/LacCer conversion is the key event leading to signaling by the elastin receptor complex. As a consequence, we propose that LacCer is an early messenger for this receptor.

## Introduction

Elastin is the extracellular matrix protein responsible for the elasticity of tissues where resilience is required such as skin, large arteries or lung [1]. This protein can be degraded in elastin peptides. Unlike elastin, some of these fragments (i.e. those presenting the GXXPG pattern) exhibit a strong biological activity [2]. These elastin peptides, or elastokines, are produced during various physiological processes following the action of elastases.

Elastin peptides regulate several biological functions such as chemotaxis [3], [4], proliferation [5], proteases synthesis [6], [7] in normal and tumor cells suggesting that they are involved in tumor progression [2] and vascular pathologies [8].

The biological activity of elastin peptides is regulated by their binding to the elastin receptor complex. In human, this complex comprises three sub-units: a peripheral protein of 67 kDa called elastin binding protein (EBP, accession number P16279) and two membrane-associated proteins, protective protein/cathepsin A (PPCA, accession number P10619) and neuraminidase-1 (Neu-1, accession number Q99519) of 55 kDa and 61 kDa respectively [9]. EBP is an enzymatically spliced variant of the lysosomal β-galactosidase (β-Gal, EC 3.2.1.23). Elastokines binding on EBP triggers the elastin receptor complex association and induces signal transduction whereas occupancy of EBP galactolectin site by galactosides causes elastin peptides release, dissociation of the complex and signal loss [2].

We have recently shown that elastin peptides binding to EBP leads to Neu-1 activation and that the induction of this sialidase activity is required for signal propagation and further induction of the extracellular signal-regulated kinase 1/2 (ERK 1/2) pathway [9], [10]. However, it has to be emphasized here that the substrates desialylated by Neu-1 remain unknown.

Neu-1 is present at the plasma membrane but it is also located in lysosomes where it is associated to β-Gal and PPCA. In the lysosome, PPCA protects β-Gal and Neu-1 from intralysosomal digestion independently of its serine-protease activity [11]. Neu-1 is a member of the sialidase family and catalyzes the removal of sialic acids from the sugar chains of glycoproteins and glycolipids [12], [13]. Seyrantepe and co-workers [13] have shown that the glycosphingolipid N-acetylneuraminic-α (2-3)-galactosyl-β (1-4)-glucosyl-β (1-1’)-ceramide acid, or GM_3_ ganglioside, is a substrate of Neu-1.

Gangliosides are sialic acid-containing glycosphingolipids found in the outer leaflet of the plasma membrane of vertebrate cells [14]. Gangliosides are amphiphilic molecules consisting of an oligosaccharidic chain of variable length and complexity bound to a ceramide anchor. These molecules belong to the glycosphingolipid family and are characterized by the presence of one or more sialic acid residues [15]. Gangliosides are involved in cellular interactions and in signal transduction [16]. Lactosylceramide (LacCer), GM_3_ ganglioside precursor, is involved in fibroblast proliferation [17], ERK 1/2 activation in smooth muscles cells [18] and angiogenesis [19].

Lipid rafts are highly organized plasma membrane microdomains enriched in cholesterol, glycosphingolipids and transmembrane proteins [20]. Within rafts, glycosphingolipids are specifically enriched at the exoplasmic leaflet while glycerolipids reside in the cytoplasmic leaflet and cholesterol in the inner spaces [21]. Rafts are important signaling platforms and numerous signal transduction schemes have been linked to their presence [20], [22].

In this study, we show that, following elastin peptides binding on EBP, the Neu-1 sub-unit of the elastin receptor complex converts the GM_3_ ganglioside to LacCer and that this conversion leads to intracellular signaling. As a consequence, we propose that the Neu-1-driven GM_3_/LacCer conversion occurring in rafts after treatment with elastin peptides is the key element of elastin signaling in cells.

## Methods

### Ethics statement

Human skin fibroblasts were established from explants of human adult skin biopsies obtained from informed healthy volunteers (aged 21–49 years) who have given their written consent. The experiments were conducted according with the recommendations of "Le Centre National de la Recherche Scientifique" (CNRS, France) which has specifically approved this study.

### Reagents

Elastin peptides were prepared as described previously [7]. Briefly, insoluble elastin was prepared from bovine *ligamentum nuchae* by hot alkali treatment. Its purity was assessed by comparing its amino acid composition to that predicted from the elastin gene product. Soluble elastin peptides were further obtained from insoluble elastin by organo-alkaline hydrolysis. This was achieved using 1 M KOH in 80% aqueous ethanol [23]. The obtained mixture of elastin peptides is termed κ-elastin (kE) and exhibits the same biological properties as physiological elastin hydrolysates obtained using elastase [7]. Dulbecco's Modified Eagle's Medium (DMEM) was purchased from Invitrogen (Cergy Pontoise, France). Fetal calf serum (FCS) was obtained from Pan Biotech GmbH distributed by Dutscher (Brumath, France). Methyl-β-cyclodextrin (MCD), protease inhibitor cocktail, MTT and lactose were purchased from Sigma-Aldrich (Saint Quentin Fallavier, France). [1-^3^H] sphingosine, glycosphingolipid biosynthesis precursor, was obtained from Perkin Elmer (Courtaboeuf, France). HPTLC sheets were purchased from Merck Biosciences distributed by VWR (Strasbourg, France). BCAssay Protein Quantitation Kit was purchased from Interchim (Montluçon, France). D-threo-1-phenyl-2-decanoylamino-3-morpholino-4 propanol (D-PDMP) was obtained from Matreya, Biovalley (Marne la Vallée, France). AlexaFluor 488-cholera toxin B sub-unit and streptavidin-AlexaFluor 568 were purchased from Molecular Probes (distributed by Invitrogen).

### Antibodies

The rabbit polyclonal anti-EBP antibody was raised against the EBP specific segment coupled to a GST carrier. IgG were purified and those raised against GST were removed by GST-sepharose affinity. Monoclonal anti-β-actin, anti-mouse and mouse anti-IgG horseradish peroxydase-coupled antibodies were purchased from Sigma-Aldrich. Monoclonal anti-GM_3_ antibody (ref M2590) was obtained from Cosmo Bio (Tokyo, Japan). Anti-cholera toxin B sub-unit antibody was purchased from Molecular Probes. Polyclonal anti-phospho-ERK1/2 (T202/Y204), anti-ERK 1/2 and horseradish peroxidase-coupled anti-rabbit antibodies were obtained from Cell Signaling Technology Inc. (Beverly, MA, distributed by Ozyme, Saint Quentin en Yvelines, France). Biotin-anti-IgG rabbit antibody was purchased from Jackson ImmunoResearch distributed by Interchim. FITC-anti-IgM control antibody was purchased from Miltenyi Biotec (Paris, France). FITC-anti-CD17 antibody (anti-lactosylceramide) was obtained from Ancell distributed by Covalab (Villeurbanne, France). Polyclonal anti-Neu-1 antibody was purchased from Santa Cruz Biotechnology (distributed by Tebu, Le Perray en Yvelines, France). Monoclonal blocking antibodies directed against α_v_β_3_ and galectin-3 (Gal3) were purchased from Millipore (Molsheim, France).

### Fibroblast culture and treatments

Human skin fibroblasts were established from explants of human adult skin biopsies obtained from informed healthy volunteers (aged 21–49 years) as previously described using cell culture medium selection [24]. Cells were grown as monolayer cultures in DMEM containing 1 g/l of glucose, glutamax I and pyruvate, supplemented with 10% FCS and in the presence of 5% CO_2_. Cells at sub-cultures 4 to 8 were used. For experiments, fibroblasts were grown to sub-confluence in medium containing 10% FCS. Before stimulation, cells were incubated for 18 h in DMEM supplemented with 0.5% FCS, washed twice with PBS and incubated in serum-free DMEM with or without kE and the different inhibitors, antagonists and blocking antibodies for the indicated times. Cell survival following D-PDMP treatment was evaluated using MTT assay following manufacturer's instruction. The different modulators were present in the cell culture media during the stimulation. Cell stimulation was stopped by adding ice-cold phosphate buffered saline (PBS) containing 50 µM Na_3_VO_4_.

### Immunocytochemistry

Cells cultured on 24-well plates containing cover glasses were washed with PBS (pH 7.4) and fixed 5 min at room temperature with 4% formaldehyde (w/v) in PBS. After PBS washings, the cell layer was saturated with 10% normal goat serum (NGS) in PBS during 30 min at room temperature. The anti-EBP antibody (1/10 dilution) was incubated for 1 h. A Biotin-anti-IgG rabbit antibody (1/50 dilution) in 3% NGS was incubated at room temperature during 30 min. Cells were incubated for 30 min with streptavidin-AlexaFluor 568. Cover glasses were incubated with AlexaFluor 488- cholera toxin B sub-unit diluted to 1/1000 in DMEM for 10 min at 4°C in darkness. Cells were washed and an anti-cholera toxin B sub-unit antibody (1/200) was added during 15 min at 4°C. Cells were observed with a Bio-Rad 1024 S confocal microscope (Bio-Rad, Marne-la-Vallée, France).

### Lipid analysis

Fibroblasts were incubated in the presence of 0.4 µCi [1-^3^H] sphingosine. After 2 h of incubation (pulse), the medium was removed. Cells were washed with PBS and incubated for 48 h (chase) with DMEM devoid of radioactive precursors in the presence of 10% FCS [25]. At the end of the chase, the medium was carefully collected and cells were harvested by scrapping with a rubber policeman. Cell suspensions were mixed with a chloroform/methanol (2∶1, v/v) mixture and centrifuged (5000 *g*, 10 min, 4°C). Lipids were analyzed by HPTLC using a chloroform/methanol/0.2% aqueous CaCl_2_ (60∶40∶9, v/v/v) mixture to separate aqueous phase and a chloroform/methanol/water (55∶20∶3, v/v/v) mixture, to isolate organic phase lipids. After 90 min, HPTLC sheets were revealed with iodine, covered by a KODAK MS film and stored at -80°C in a cassette.

### Flow cytometry

After stimulation, fibroblasts were washed with ice-cold PBS containing 50 µM Na_3_VO_4_ and harvested using PBS EDTA 10 mM at 4°C. Cells were centrifuged (400 *g*, 10 min, 4°C), washed with PBS, then incubated with FITC-anti-CD 17 antibody (anti-lactosylceramide) or FITC-anti-IgM control (1/25 dilution) for 20 min at room temperature in darkness. Cells were then washed, centrifuged and fixed with 500 µl of 1% formaldehyde before being analyzed with a FACS Calibur cytometer (Becton Dickinson, Le Pont de Claix, France).

### Western-blotting

Fibroblasts (10^6^) were washed twice in ice-cold PBS containing 50 µM Na_3_VO_4_, scrapped and sonicated in lysis buffer (PBS, pH 7.4, 0.5% Triton X-100, 80 mM glycerophosphate, 50 mM EGTA, 15 mM MgCl_2_, 1 mM Na_3_VO_4_, and protease inhibitor cocktail). Insoluble material was removed by centrifugation (20000 *g*, 20 min, 4°C). Protein concentrations were determined by BCAssay Protein Quantitation Kit. Equal amounts of proteins were heated for 5 min at 100°C in Laemmli sample buffer, resolved by SDS-PAGE under reducing conditions and transferred to nitrocellulose membranes. The membranes were placed in blocking buffer (5% (w/v) nonfat dry milk in Tris-buffered saline/Tween 20 (50 mM Tris, pH 7.5, 150 mM NaCl, and 0.1% (v/v) Tween 20)) for 1 h at room temperature and incubated overnight at 4°C with anti-phospho-ERK1/2 (T202/Y204), anti-ERK 1/2 (1/1000), anti-Neu-1 (1/200) or anti-β-actin (1/4000) antibodies. After five washings with Tris-buffered saline/Tween 20, membranes were incubated for 1 h at room temperature in the presence of horseradish peroxidase-coupled anti-rabbit antibodies or horseradish peroxidase-coupled anti-mouse antibodies (1/4000 and 1/10000 in blocking buffer, respectively). Immuno-complexes were detected by chemiluminescence. In order to evaluate ERK 1/2 phosphorylation, observed bands were analyzed using the densitometric PhosphorAnalyst software (Bio-Rad).

### Neu-1 siRNA gene silencing

Fibroblasts were grown on 6-well plates to 70% confluency in DMEM containing 1 g/l of glucose, glutamax I and pyruvate, supplemented with 10% FCS and in the presence of 5% CO_2_. For siRNA transfection, we used the predesigned silencer Neu-1 siRNA from Ambion (5′-GGCGAGGAAAAUGUCCUCATT-3′ (forward), 5′-UGAGGACAUUUUCCUCGCCTC-3′ (reverse); ID: 8481, distributed by Applied Biosystems, Courtaboeuf, France) as they are very efficient Neu-1 siRNA [9]. The negative control siRNA (SNC siRNA) was also purchased from Ambion (cat. n°AM4611). Cells were trypsinized, centrifuged (400 *g*, 10 min, 20°C), then 400 000 cells were nucleofected. After nucleofection (Nucleofector, Amaxa Biosystems, Germany) with Neu-1 or SNC siRNA (100 pmol), fibroblasts were cultured on 6-well plates for 48 h and cell extracts were analyzed by Western-blotting as described above.

### Statistical analysis

All experiments were performed three times in triplicate. Results are expressed as mean +/− S.E.M. Comparison between groups were made using Student's *t* test. The results were considered significantly different at *p*<0.05.

## Results

### EBP and lipid rafts colocalize at the plasma membrane

**Figure 1:**
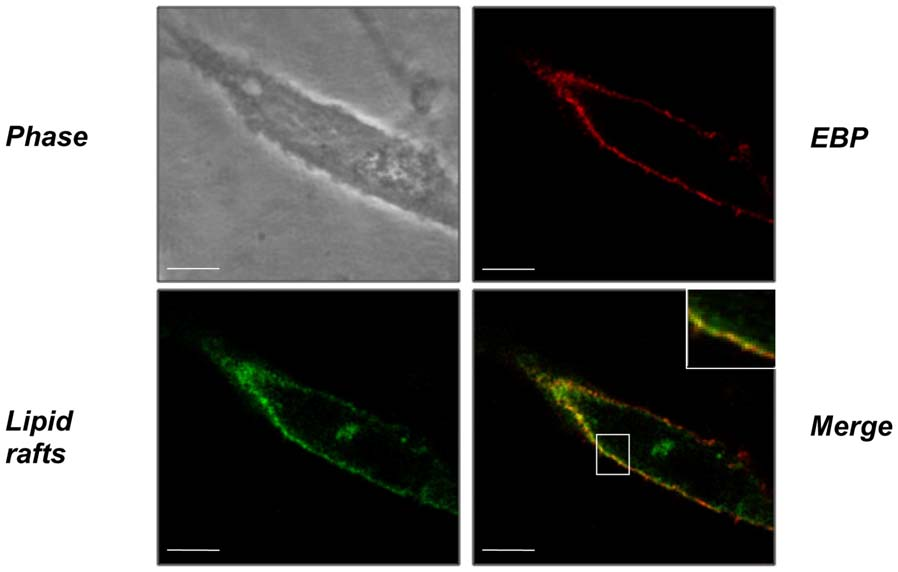
EBP and lipid rafts are colocalized at the plasma membrane. EBP and lipid rafts localization were analyzed using confocal microscopy as described in the Methods section. EBP appears as a red staining, lipid rafts as a green staining and the colocalization as a yellow staining. The scale represents 10 µm.

Among glycosphingolipids which are concentrated in lipid rafts, the GM_3_ ganglioside has been described as a substrate of Neu-1 [13], [15], [20]. As a consequence, we first checked the relative localization of EBP and of these microdomains. This was achieved by a double immunocytochemical staining followed by fluorescence microscopy imaging (Fig. 1). We observed a homogeneous distribution of EBP and lipid rafts on the plasma membrane of human dermal fibroblasts and the merge of the obtained images indicated that these entities colocalized.

### Lipid rafts integrity is required for elastin peptide-induced signal transduction

**Figure 2:**
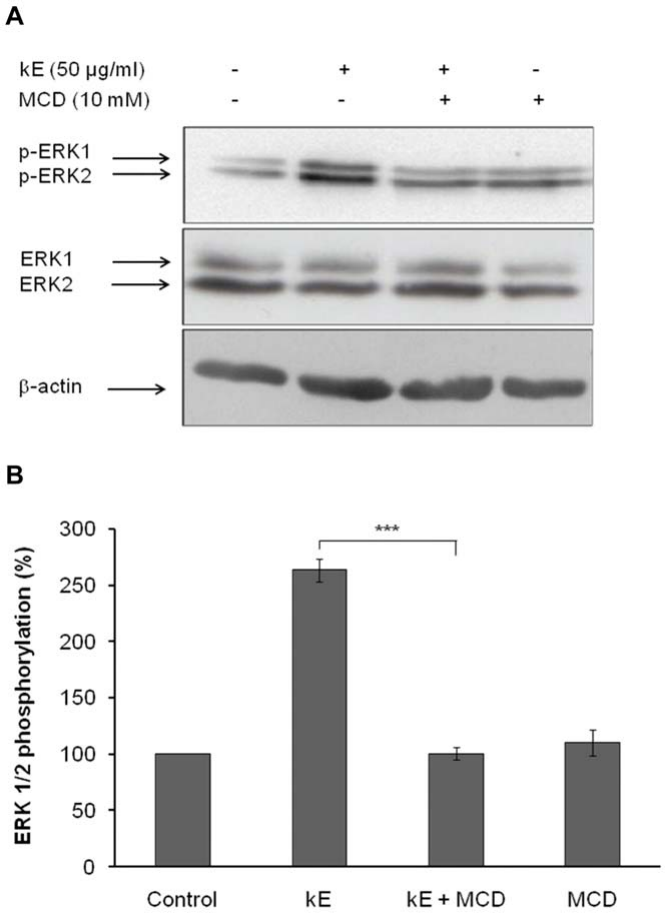
Lipid rafts integrity is required for elastin peptide-induced signal transduction. **A**) Fibroblasts were incubated for 30 min with 10 mM MCD then treated with 50 µg kE/ml for 30 min. Cellular extracts were analyzed by anti-phospho-ERK 1/2 (T202/Y204) and anti-ERK 1/2 Western-blots. **B**) Densitometric analysis: ***, *p*<0.001.

Lipid rafts are plasma membrane microdomains specialized in signaling. They are enriched in cholesterol and this lipid is necessary for their function. MCD is a derivative of a glucose cyclic oligomer with a lipophilic property. It has the ability to extract cholesterol from membranes and is therefore commonly used to study the role of rafts in biological processes [20]. Consequently, cholesterol depletion using MCD selectively occurs in rafts and pathways inhibited by such a treatment are defined as raft-dependent [26]. In fibroblasts, elastin peptides induce ERK 1/2 activation through the elastin receptor complex [9]. In order to evaluate the role of these structures in elastin peptide-induced signaling, we depleted the cholesterol from rafts using MCD and tested afterwards the ability of elastin peptides to trigger ERK 1/2 activation (Fig. 2).

In the presence of elastin peptides, a 2.6 fold increase in ERK 1/2 activation was observed. This effect was totally lost when cells had been formerly cholesterol-depleted by MCD. These results strongly suggested that lipid rafts were important for signaling by the elastin receptor complex.

### Gangliosides are essential for elastin peptide-induced signal transduction

**Figure 3:**
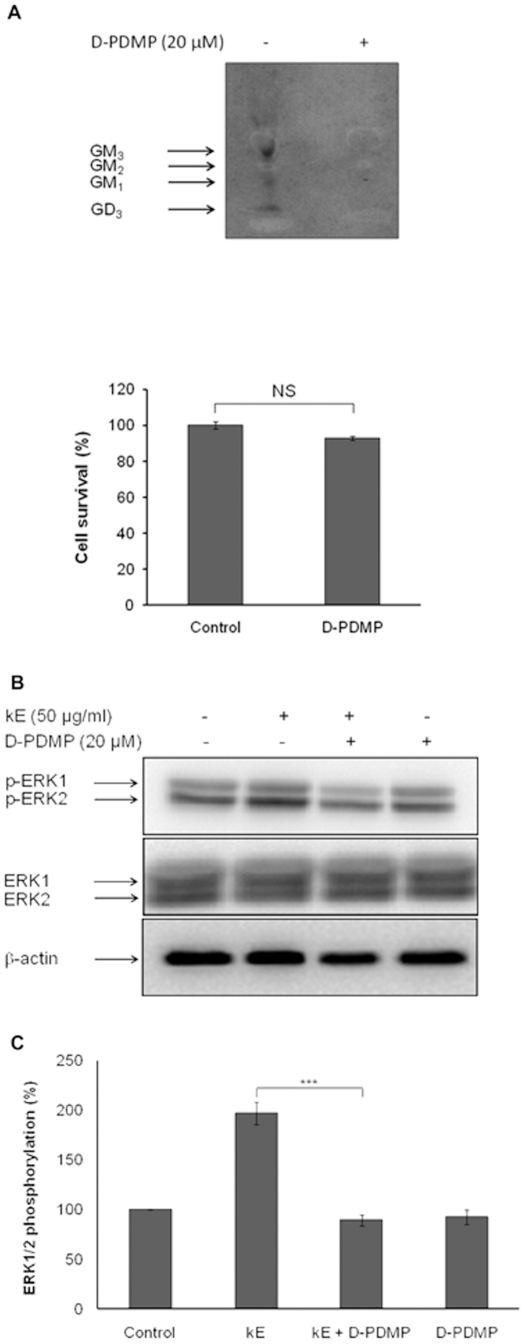
Gangliosides are essential for elastin peptide-induced signal transduction. **A**) *Upper panel*: Fibroblasts were incubated for 2 h with [1-^3^H]-sphingosine. The radioactive staining of glycolipids was realized for 48 h. Glycosphingolipids depletion was achieved by incubating cells for 3 days with 20 µM D-PDMP. After extraction, lipids were resolved by HPTLC and the plates were put in presence of a radiographic film during one month at −80°C, and then revealed. *Lower panel*: cell survival evaluated using MTT assay after D-PDMP incubation. **B**) Cells were incubated for 3 days with 20 µM D-PDMP. Fibroblasts were then treated with 50 µg kE/ml for 30 min. Cellular extracts were analyzed by anti-phospho-ERK 1/2 (T202/Y204) and ERK 1/2 Western-blots. The emergence of a third band in ERK1/2 Western-blots is due to an electrophoretic shift because of its phosphorylation. **C**) Densitometric analysis: ***, *p*<0.001.

Glycosphingolipids and especially gangliosides are major and specific components of lipid rafts. They modulate several aspects of signal transduction processes [15]. In order to determine the role of these glycosphingolipids in elastin peptide-induced signaling, we incubated cells with D-PDMP, an UDP-glucose/galactose transferase inhibitor [27], for several days so as to totally deplete these lipids from cell membranes. The analysis of fibroblasts gangliosides (Fig. 3A) evidenced large GM_3_ and GD_3_ proportions as well as GM_2_ and GM_1_. D-PDMP totally blocked gangliosides synthesis without inducing significant cell death.

Fibroblasts lacking gangliosides totally failed to induce ERK 1/2 activation after elastin peptides treatment (Fig. 3B and 3C). This result suggests that the gangliosidic component of lipid rafts played an essential role in elastin peptide-induced cell signaling.

### GM_3_ is converted to LacCer following elastin peptides treatment

**Figure 4:**
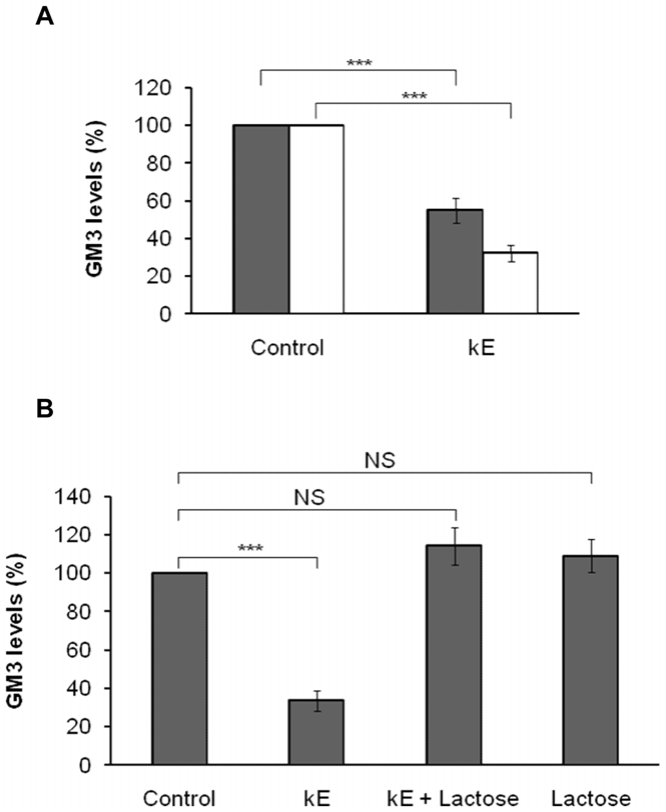
EBP is involved in the kE-induced GM_3_ levels decrease. **A**) Fibroblasts were incubated for 2 h with [1-^3^H]-sphingosine, PBS washed, and radioactive staining of glycolipids was performed for 48 h. Cells were then stimulated with or without 50 µg kE/ml during 5 (dark grey) or 30 min (white). After extraction, lipids were separated by HPTLC and the plates were put in the presence of a radiographic film during one month at −80°C and revealed. GM_3_ was identified comparing its migration rate to that of controls. Densitometric analysis: ***, *p*<0.001. **B**) The radioactive staining and the stimulation have been performed as in A. Lactose (1 mM), an EBP antagonist, was preincubated for 3 h before stimulation with kE (50 µg/ml, 30 min). After extraction, lipids were separated by HPTLC and the plates were put in presence of a radiographic film during one month at −80°C, and revealed. GM_3_ was identified by comparing its migration rate to that of controls. Densitometric analysis: NS, not significant; ***, *p*<0.001.

**Figure 5:**
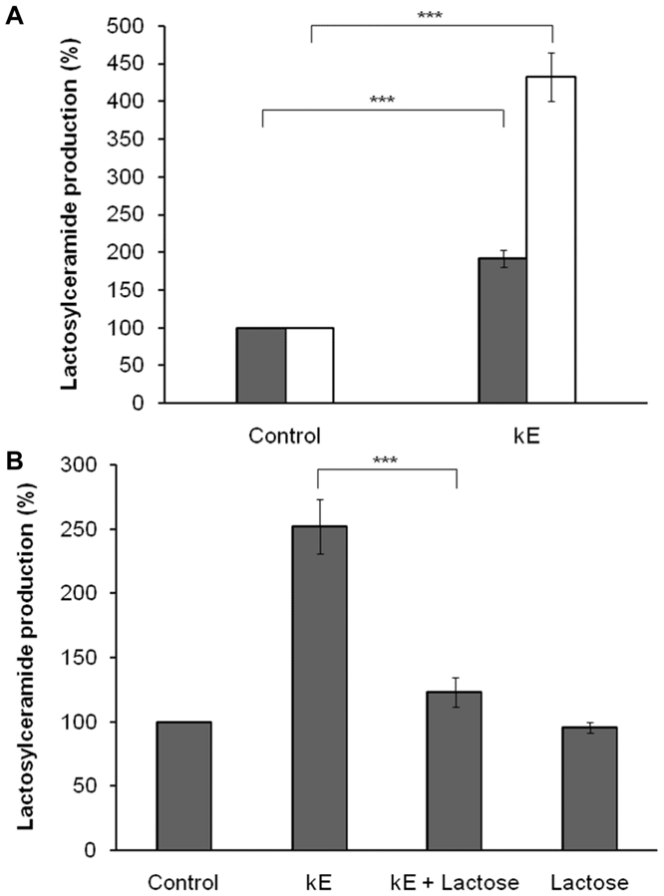
EBP is involved in the kE-induced LacCer production. **A**) Radioactive staining and stimulation were performed as described in Figure 5 caption. LacCer was identified by comparing its migration rate to that of controls. Densitometric analysis: ***, p<0.001. **B**) Lactose (1 mM) was preincubated for 3 h before stimulation with 50 µg kE/ml for 30 min. After extraction, lipids were separed by HPTLC and the plates were put in presence of a radiographic film during one month at −80°C, and then revealed. LacCer was identified comparing its migration rate to that of controls. Densitometric analysis: ***, *p*<0.001.

GM_3_ ganglioside is a substrate of Neu-1 [13]. Its desialylation by Neu-1 produces LacCer, a second messenger able to activate ERK 1/2 pathway [18]. In order to evaluate the influence of kE on GM_3_ and LacCer levels, cellular gangliosides were labelled with [1-^3^H]-sphingosine and analyzed by HPTLC (Fig. 4A and 5A) after cells were treated or not with elastin peptides. Our results showed an inverse correlation between the GM_3_ and LacCer levels. At 5 min stimulation, kE induced a 45% decrease of GM_3_ level and a 92% increase of LacCer level. This effect was amplified at 30 min stimulation, showing a 68% decrease of GM_3_ level and a 332% increase of LacCer level. These observations suggested that GM_3_ was converted to LacCer after elastin peptides treatment.

### GM_3_/LacCer conversion is regulated by the elastin receptor complex

In order to determine the role of the elastin receptor complex in this conversion, similar experiments were performed in the presence of lactose, an EBP antagonist (Fig. 4B and 5B). We noticed that the elastin peptide-induced GM_3_ decrease could not be observed anymore in the presence of lactose. Moreover, the formerly observed LacCer production increase was also blocked when lactose was present in the medium.

As lactose inhibited elastin receptor complex-induced signaling, these results strongly suggested that GM_3_/LacCer conversion occurred when elastin peptides were bound on the EBP sub-unit of the elastin receptor complex.

**Figure 6:**
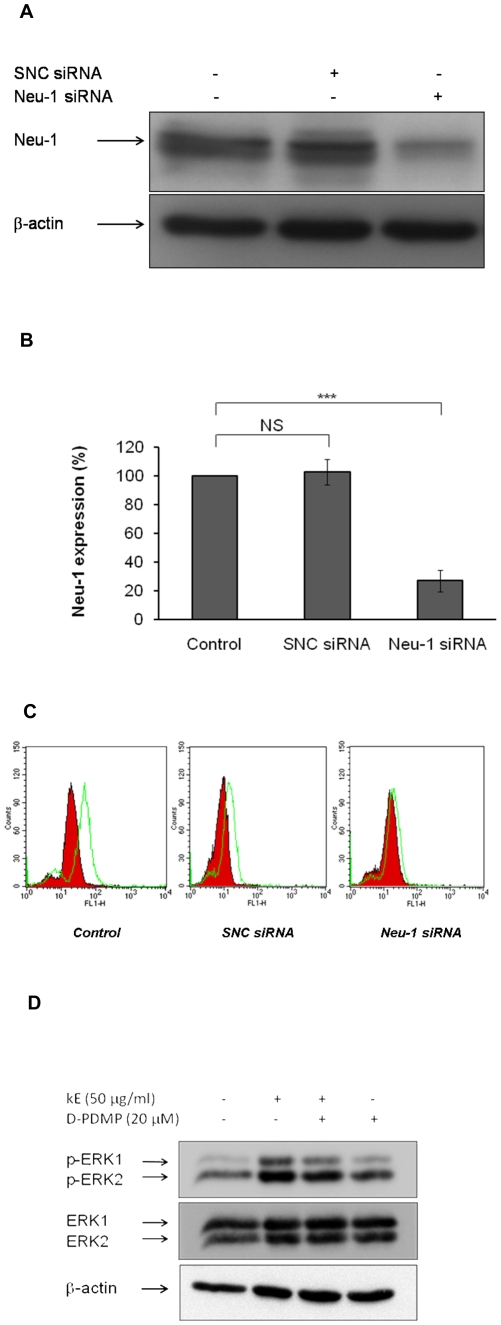
Neu-1 is involved in GM_3_/LacCer conversion. **A**) Cellular extracts from untransfected fibroblasts or cells transfected with SNC siRNA or Neu-1 siRNA were analyzed by anti-Neu-1 Western-blot. **B**) Densitometric analysis: NS, not significant; ***, *p*<0.001. **C**) Untransfected fibroblasts (control) and fibroblasts transfected with SNC siRNA or Neu-1 siRNA were stimulated with 50 µg/ml of kE. They were stained with or without an FITC-anti-LacCer antibody (1/25 dilution) then analyzed by flow cytometry. The red line represents the control and the green one corresponds to the stimulation with kE (50 µg/ml). **D**) Fibroblasts were pre-incubated with D-PDMP (20 µM) for 30 min prior to stimulation with kE (50 µg/ml, 30 min). Cell extracts were analyzed by anti-phospho-ERK 1/2 (T202/Y204) and anti-ERK 1/2 Western-blots.

It has been demonstrated that elastin peptides promote Neu-1 activity which is responsible for signal transduction by the elastin receptor complex [9]. In order to demonstrate Neu-1 involvement in the observed GM_3_/LacCer conversion, sialidase silencing was performed by nucleofection. When cells were transfected with Neu-1 siRNA, Neu-1 expression was reduced by 73% in fibroblasts (Fig. 6A and 6B). As shown by flow cytometry data (Fig. 6C), a 5 min stimulation with kE induced LacCer production in both control cells (+128%) and in SNC siRNA-transfected cells (+112%). However, no fluorescence shift was observed for stimulated Neu-1 siRNA-transfected cells demonstrating that LacCer was not produced. These results supported our view that LacCer was generated by Neu-1 following elastin peptides treatment.

**Figure 7:**
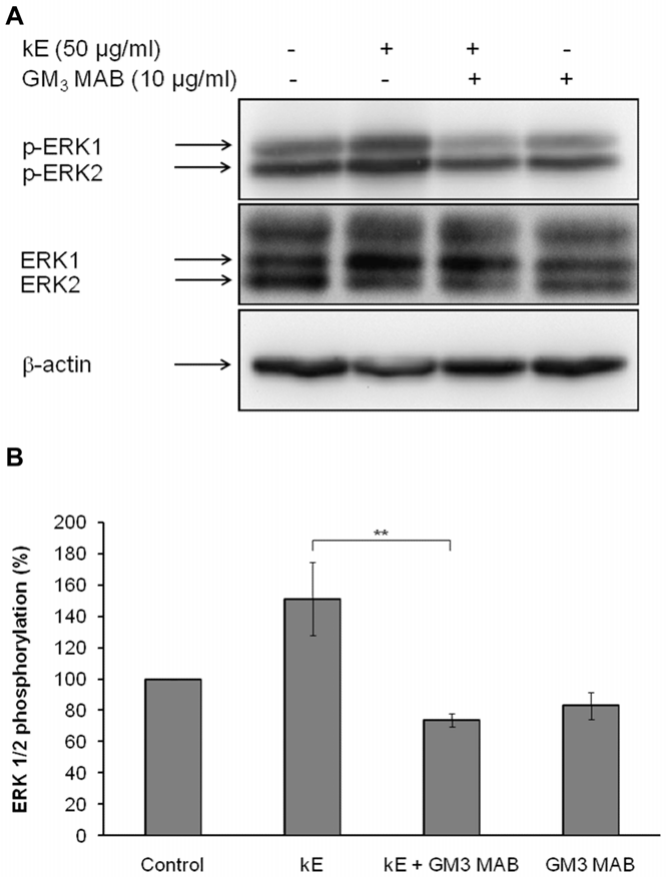
GM_3_ plays a substrate role for the elastin receptor complex. **A**) Fibroblasts were incubated for 30 min with 10 µg/ml anti-GM_3_ antibody then treated with 50 µg/ml kE for 30 min. Cell extracts were analyzed by anti-phospho-ERK 1/2 (T202/Y204) and anti-ERK 1/2 Western-blots. The emergence of a third band in ERK1/2 western-blots is due to an electrophoretic shift because of its phosphorylation. **B**) Densitometric analysis: **, *p*<0.01.

In order to show that the GM_3_ ganglioside could be the Neu-1 substrate giving rise to LacCer production, we used a monoclonal anti-GM_3_ blocking antibody [28] and tested its effect on the ability of cells to induce ERK 1/2 activation in the presence of elastin peptides. As shown in Figure 7, the presence of the anti-GM_3_ antibody totally blocked elastin peptide-triggered ERK 1/2 activation suggesting that elastin receptor complex signaling was possible only when GM_3_ was available. Finally, to exclude the possible involvement of LacCer neosynthesis in the elastin peptide-induced ERK1/2 activation, we used D-PDMP to block lactosylceramide synthase activity as previously described [19]. Our results (Fig. 6D) showed that LacCer neosynthesis is not required for elastin peptide-induced signaling.

### LacCer mimics elastin peptides effects

LacCer has been described as a second messenger able to activate ERK 1/2 pathway in smooth muscle cells [18], fibroblasts [17] and endothelial cells [19]. We thus decided to analyze the potential early messenger role of LacCer in our system.

**Figure 8:**
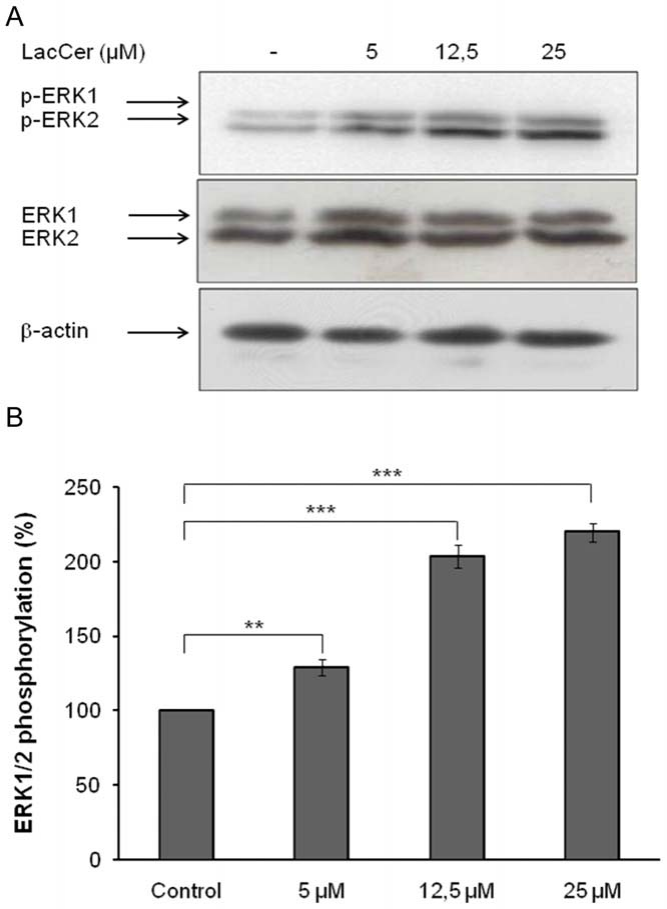
LacCer triggers ERK 1/2 activation. **A**) Fibroblasts were incubated with different concentrations of LacCer for 30 min. Cell extracts were analyzed by anti-phospho-ERK 1/2 (T202/Y204) Western-blot. **B**) Densitometric analysis: **, *p*<0.01; ***, *p*<0.001.

Cells were treated with different concentrations of exogenous LacCer and its influence on ERK 1/2 activation was analyzed by Western-blot (Fig. 8). The results indicated a dose-dependent ERK 1/2 activation with a maximal effect at 12.5 µM LacCer. Thus, exogenous LacCer could reproduce the effects of elastin peptides treatment.

**Figure 9:**
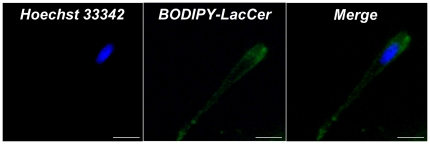
Exogenous LacCer penetrates into the plasma membrane. Fibroblasts were incubated with 12.5 µM BODIPY-LacCer (λ_excitation_ = 505 nm; λ_emission_ = 511 nm) for 30 min at 4°C followed by washings with defatted-BSA to remove any fluorescent lipid remaining at the plasma membrane as described previously [38]. Cells were fixed then analyzed by fluorescence imaging. **Hoechst 33342**: nucleus staining by Hoechst 33342 (λ_excitation_ = 340 nm; λ_emission_ = 465 nm); **BODIPY-LacCer**: LacCer staining; **Merge**: superposition of nuclear and 12.5 µM BODIPY-LacCer stainings. The scale represents 10 µm.

In order to confirm that LacCer effect was due to cell penetration, fibroblasts were incubated with BODIPY-labelled LacCer (Fig. 9). Fluorescence microscopy observation revealed a membrane staining and a cellular penetration of exogenous LacCer. As a consequence, we concluded that LacCer generated at the plasma membrane following GM_3_ conversion could explain ERK 1/2 activation.

## Discussion

The elastin receptor complex is found at the surface of numerous cell types and its involvement in physiopathological processes makes it a potential pharmacological target. The activity of its Neu-1 sub-unit is responsible for its signaling transduction capability but the substrates involved in this mechanism still remain unknown.

Using immunocytochemical methods, we observed that lipid rafts and EBP were colocalized in human dermal fibroblasts (Fig. 1). This result suggested that these membrane microdomains could be important to elastin receptor complex function.

We therefore used MCD to sequester cholesterol and deplete lipid rafts in our cells. MCD treatment totally blocked kE-induced ERK 1/2 activation (Fig. 2). This result demonstrated that intact lipid rafts were required for proper elastin receptor signaling. Lipid rafts are involved in numerous cellular processes and many studies have shown their role in signal transduction [20]. Indeed, lipid rafts are crucial to EGF receptor [29], insulin receptor [30], or integrins [31] functioning. Our data suggest that the elastin receptor complex is another lipid raft-dependent receptor.

Neu-1 activity is responsible for the elastin receptor complex signaling [9] and gangliosides, glycosphingolipids signaling regulators found in lipid rafts, are among its substrates [13]. In order to address their importance in elastin receptor-mediated signal transduction, we used D-PDMP to block their biosynthesis in fibroblasts and checked the ability of these cells to respond to elastin peptides stimuli. Consistent with the work of Makino and co-workers [27], our results show that D-PDMP-treated cells are devoid of gangliosides (Fig. 3A). Importantly, cell survival is not affected. In the presence of elastin peptides, these cells failed to activate the ERK 1/2 pathway (Fig. 3B and 3C). This point suggested that the gangliosidic component of lipid rafts was crucial for elastin receptor-induced signaling.

Glycosphingolipids and their metabolites are important in various biological processes. Iwamoto and co-workers have demonstrated the essential role of glycosphingolipids in RANKL-induced osteoclastogenesis [32]. The authors have shown that D-PDMP strongly reduced the expression of RANK induced by macrophage colony-stimulating factor. This effect was explained by the inactivation of ERK 1/2. Our data are in agreement with this signaling scheme.

Among gangliosides, we focused on GM_3_, a possible substrate of Neu-1 [13] because its desialylation leads to LacCer production, a second messenger regulating ERK 1/2 activation [18]. HPTLC analysis of GM_3_ and LacCer indicated that kE induced a decrease of GM_3_ level correlated to an increase of LacCer level. This effect was maximum at 30 min (Fig. 4A and 5A), the time at which maximum elastin peptide-induced ERK 1/2 activation is observed [10]. This finding supported our hypothesis that kE treatment leads to GM_3_/LacCer conversion eventually resulting in ERK 1/2 induction.

In order to determine if this conversion could involve the elastin receptor complex, the previous experimentations were performed in the presence of lactose, an EBP antagonist. The decrease of the GM_3_ content and the increase of LacCer production occurring after elastin peptides treatment were blocked in the presence of lactose (Fig. 4B and 5B) suggesting that the EBP sub-unit of the elastin receptor complex was involved in the GM_3_/LacCer conversion mechanism. It has been suggested that Gal3, a membrane receptor of galactolectin type, and α_v_β_3_ integrin could also bind elastin peptides [33]. Nevertheless, Gal3 and α_v_β_3_ integrin involvement in this mechanism can be excluded because our experiments with monoclonal anti-α_v_β_3_ and anti-galectin-3 blocking antibodies demonstrated that these receptors were not involved in kE-induced ERK1/2 activation (Table 1).

**Table 1:** Influence of anti-α_v_β_3_ and anti-galectin-3 blocking antibodies on ERK1/2 activation.

	ERK1/2 activity (%)
Control	100
kE	141,26±1,93
kE + αvβ3 mAb	140,58±2,31
kE + Gal3 mAb	140,28±2,07
αvβ3 mAb	99,16±1,02
Gal3 mAb	98,72±1,39

Fibroblasts were pre-incubated with anti-α_v_β_3_ or anti-galectin-3 blocking antibodies (5 µg/ml) for 30 min before stimulation or not with kE (50 µg/ml; 30 min). ERK1/2 activity was assessed by Western-blots.

The elastin receptor complex involvement in LacCer production following elastin peptides treatment was quantitated by flow cytometry (Fig. 6C). We observed that fibroblasts in which the Neu-1 sub-unit of the complex was silenced could no more initiate LacCer production whereas fibroblasts transfected with a control siRNA retained this possibility. These data strongly support our view that the GM_3_/LacCer conversion occurring following elastin peptides treatment relies on Neu-1 sialidase activity.

In order to confirm the substrate role of GM_3_ ganglioside, we used a blocking monoclonal antibody directed against GM_3_ [28] and observed that cells treated with this antibody could not respond to the presence of elastin peptides in the medium (Fig. 7). As a consequence, we concluded that GM_3_ is the substrate of Neu-1.

We checked the messenger role of LacCer in elastin receptor complex signaling by adding exogenous LacCer to fibroblasts in culture. We showed that LacCer presence leads to an increase in ERK 1/2 activation in a dose-dependent manner (Fig. 8) with a maximal concentration at 12.5 µM. Our results are in agreement with those of Chatterjee and co-workers [18] who observed that 10 µM LacCer induced ERK 1/2 activation in smooth muscle cells. To verify that LacCer action was correlated to its penetration into the plasma membrane, we used BODIPY-LacCer. [34]. Our observations confirmed cell penetration of this lipid (Fig. 9), explaining its bioactivity.

As a consequence, we propose that LacCer produced in the plasma membrane by Neu-1-mediated GM_3_ conversion is an early messenger of the elastin receptor complex. Further, we feel that GM_3_/LacCer conversion could be a fundamental event of elastin peptides biology as it could explain most aspects of their biological activities. Indeed, a strong correlation exists between elastin peptides biological effects and LacCer activities such as regulation of cell proliferation [5], [17], angiogenesis [19], [35], activation of ERK 1/2 [9], [18]. Moreover, signaling by this glycosphingolipid perfectly overlaps elastin peptide-derived signaling such as Ras activation [36], [37], cytosqueleton reorganization and Src activation [35], [37], [38].

Importantly, our discovery that LacCer is an early messenger of the elastin peptides receptor complex could have strong implications in ageing. Robert and co-workers [39] have underlined that a large number of receptors functions are lost due to ageing. The elastin receptor complex is one of them. With age, an uncoupling of this receptor from its signaling pathways is observed although its affinity for its cognate ligands remains unchanged in aged as compared to young cells. We propose that alterations of membrane lipid compositions, notably in rafts, could explain the inability of aged cells to transduce signals originating from the elastin receptor complex. Indeed, membrane gangliosides composition is largely age-dependent as old subjects possess less GM_3_ than young ones [40]. Consequently, stimulation of aged cells by elastin peptides would lead to less LacCer production, thereby explaining the apparent loss of function for their elastin receptor complex.

In conclusion, our work shows that the elastin receptor complex transduces its signals through an original mechanism involving Neu-1-mediated GM_3_/LacCer conversion. Additionally, our proposal could explain most elastin peptides biological effects and their physiopathological consequences. These new data let us foresee new approaches to block or strengthen elastin peptides contribution in pathological or pharmacological situations.
